# *HECTD2*, a candidate susceptibility gene for Alzheimer's disease on 10q

**DOI:** 10.1186/1471-2350-10-90

**Published:** 2009-09-15

**Authors:** Sarah E Lloyd, Martin Rossor, Nick Fox, Simon Mead, John Collinge

**Affiliations:** 1 MRC Prion Unit, Department of Neurodegenerative Disease, UCL Institute of Neurology, London, WC1N 3BG, UK; 2 Department of Neurodegenerative Disease, UCL Institute of Neurology, London, WC1N 3BG, UK

## Abstract

**Background:**

Late onset Alzheimer's disease (LOAD) is a neurodegenerative disorder characterised by the deposition of amyloid plaques and neurofibrillary tangles in the brain and is the major cause of dementia. Multiple genetic loci, including 10q, have been implicated in LOAD but to date, with the exception of *APOE*, the underlying genes have not been identified. *HECTD2 *maps to 10q and has been implicated in susceptibility to human prion diseases which are also neurodegenerative conditions associated with accumulation of misfolded host proteins. In this study we test whether the *HECTD2 *susceptibility allele seen in prion disease is also implicated in LOAD.

**Methods:**

DNA from 320 individuals with Alzheimer's disease and 601 controls were genotyped for a *HECTD2 *intronic tagging SNP, *rs12249854 *(A/T). Groups were further analysed following stratification by *APOE *genotype.

**Results:**

The *rs12249854 *minor allele (A) frequency was higher (5.8%) in the Alzheimer's disease group as compared to the controls (3.9%), however, this was not statistically significant (P = 0.0668). No significant difference was seen in minor allele frequency in the presence or absence of the *APOE *ε4 allele.

**Conclusion:**

The common haplotypes of *HECTD2*, tagged by *rs12249854*, are not associated with susceptibility to LOAD.

## Background

Alzheimer's disease (AD) is the most common form of dementia affecting millions of people worldwide [1]. The decline in cognitive ability is accompanied by characteristic pathological changes in the brain which include deposition of extracellular amyloid plaques composed of β-amyloid (Aβ) [2] and the accumulation of intracellular neurofibrillary tangles (hyperphosphorylated tau) [3]. Mutations in three genes, β-amyloid precursor protein (*APP*), presenilin-1 (*PSEN1*) and presenilin-2 (*PSEN2*), have been found in early-onset autosomal dominant AD [4-6]. In contrast, the majority of AD cases have a later age of onset (>65 years) [1] and although heritability is high, there are multiple environmental and genetic risk factors with no clear Mendelian pattern of inheritance [7]. To date, the main genetic risk factor for late onset Alzheimer's disease (LOAD) is the apolipoprotein E gene (*APOE*) where the ε4 allele is over-represented in affected individuals (50%) compared to controls [8]. The partial penetrance for *ε*4 suggests that other genes are also likely to be important in the development of LOAD [9].

Linkage studies, and more recently genome-wide association studies (GWAS), have provided evidence to support a role for multiple genes in the aetiology of LOAD [9,10]. Difficulties in replicating findings across multiple studies still leaves considerable uncertainty in defining the genetic risk factors. Significant evidence exists for a susceptibility locus on chromosome 10q [11-14], however, there are conflicting reports on its precise location and to date no clear association to an individual gene has been demonstrated. Insulin degrading enzyme (IDE) is encoded by a gene that maps to Chr 10q and has been implicated in the breakdown and clearance of extracellular Aβ [15]. Although there is contradictory evidence for an allelic association between *IDE *and AD [16-18] a linkage study carried out in 435 multiplex AD families found significant linkage to markers adjacent to *IDE *(*D10S583 *and *D10S1671*) [11,19].

*HECTD2 *encodes an E3-ubiquitin ligase protein and maps within 1 Mb of *D10S583 *and *IDE*. *HECTD2 *has been linked to prion disease incubation time in mice and a susceptibility haplotype has been associated with human prion disease [20]. Prion diseases, like AD, are neurodegenerative disorders with a complex aetiology. Mutations in the prion protein gene (PRNP) cause the inherited prion diseases [21,22] and coding polymorphisms in the prion protein (PrP) are associated with susceptibility to acquired and sporadic Creutzfeldt-Jakob disease (CJD), kuru and influence age of onset in some inherited prion diseases [23-25]. Similarly to LOAD, acquired and sporadic CJD have a complex non-Mendelian genetic component where there is evidence for the role of multiple genes [26-29]. Prion diseases also share some of the neuropathologic hallmarks of AD and are considered to be diseases of protein misfolding where abnormal forms of PrP accumulate in the brain often in the form of amyloid plaques [21,30].

The similarities between AD, prion diseases and other neurodegenerative disorders suggest that common molecular mechanisms and pathways may be involved. The ubiquitin-proteosome system is one such example that has been implicated in the pathogenesis of several neurodegenerative diseases which show an accumulation of an abnormally folded protein including AD and prion disease [31-33]. The substrate and exact function of HECTD2 are unknown but by homology to other family members it is thought to function as an E3 ubiquitin ligase, catalysing the transfer of ubiquitin to specific proteins, thus targeting them for degradation by the proteosome. Mutations in an E3 ubiquitin ligase, parkin, are associated with forms of inherited Parkinson's disease [34].

The location of *HECTD2 *on Chr 10q, its association with another neurodegenerative proteinopathy and its involvement in the ubiquitin-proteosome pathway suggest that *HECTD2 *is a suitable candidate gene for LOAD susceptibility. The aim of this study was therefore to test whether the *HECTD2 *risk allele seen in CJD is also a susceptibility factor for non-familial AD.

## Methods

### Human samples

The clinical and laboratory studies were approved by the local research ethics committee of University College London Institute of Neurology and National Hospital for Neurology and Neurosurgery. Most of the samples were obtained with written consent from patients or next of kin, however, for archival samples, where this was not available, specific approval was obtained from the local ethics committee.

### Alzheimer's disease

Patient samples were derived from the MRC Prion Unit sample collection based on a clinician's diagnosis of AD (n = 320). The majority of samples were referred to the Unit for *APP*, *PSEN1 *or *PSEN2 *gene sequencing although not all genes were tested in all patients, because for example, some samples were referred prior to the discovery of presenilin gene mutations as causal in AD. Samples with causal mutations were excluded. Consequent upon patient ascertainment, the sample collection is enriched for unexplained early onset AD. Those of known non-white Caucasian ethnicity were excluded. Average age at time of sampling was 53, 47% were male. Ethical approval for the study was given by the University College London Hospitals NHS Trust Local Ethics Committee. *APOE *genotypes were obtained for n = 316 samples. Allele frequencies for ε2, ε3 and ε4 were 3.6%, 62.1% and 34.3% respectively. 55.1% of individuals had one or more ε4 alleles which is consistent with other reports of LOAD.

#### UK controls

116 individuals were recruited from the National Blood Service (NBS). Individuals were of white-British ethnicity and the mean of age at sampling was 34 years (range 18-64); 56% were male. DNA was extracted from whole blood. Further UK control samples (n = 485) were purchased from the European Collection of Cell Cultures (ECACC) Human Random control (HRC) DNA panels consisting of randomly selected, non-related UK Caucasian blood donors. Total number of UK controls was n = 601.

#### DNA extraction

Genomic DNA was usually extracted from peripheral blood using a Nucleon Genomic DNA Extraction Kit according to the manufacturer's instructions; for a small number of brain tissue samples we used a phenol-chloroform method. Amplified DNA, using either multiple displacement amplification (Geneservice, Cambridge, UK) or fragmentation-PCR methods (Genomeplex, Sigma), was used for a small number <10% of samples. Samples were checked for degradation on 1% agarose gel and stored at 50 ng/μl in 10 mM Tris-EDTA buffer.

#### Genotyping

For *rs12249854 *a pre-designed allelic discrimination assay was purchased from Applied Biosystems and used according to the manufacturer's instructions. All reactions were carried out in 5 μl on a 7500 Fast Real-time PCR System (Applied Biosystems) using RoxMegaMix Gold (Microzone Ltd). Cycling conditions were 95°C 5 minutes; 95°C 15 s, 60°C 60 s for 40 cycles.

### APOE genotyping

*APOE *genotyping was performed by Cfo1 restriction endonuclease digestion of PCR amplicon and size differentiation by agarose gel electrophoresis [35].

### Statistical genetics

Association was tested using the Pearson Chi-squared test (SPSS, SPSS Inc.). Additional permutation tests were carried out using PLINK [36].

## Results

We have previously shown that strong linkage disequilibrium (LD) extends across the whole of the *HECTD2 *gene and interrogation of HapMap data suggests that this block of LD does not extend into neighbouring genes [20]. It is therefore possible to tag the haplotype associated with susceptibility to prion disease with a single nucleotide polymorphism (SNP), *rs12249854 *(A/T) that occurs within intron 1 of *HECTD2. rs12249854 *was genotyped in a panel of 320 samples from patients with a definite or probable diagnosis of AD and no known mutations in *APP*, *PSEN1 *or *PSEN2*. *APOE *genotypes were available for 316 individuals and showed enrichment for the ε4 allele (55.1% of individual have one or more ε4 allele). Genotypes were compared with those of a previously genotyped UK control group (n = 601) that were ethnically matched [20].

The *rs12249854 *minor allele (A) frequency was higher (5.8%) in the AD group as compared to the controls (3.9%), however, this was not significant (P = 0.0668, Chi-squared test, allelic odds ratio = 1.5 (95% CI = 0.97-2.3)) (Table 1). In addition to looking at the allele frequencies we also looked at genotype specific effects. The AA genotype is rare and was not observed in the control group. While two AA individuals were seen in the AD group, this was not significant (P = 0.0648, Chi squared test). Because the expected minor allele frequency is low, the Chi-squared test may be unreliable therefore we implemented 1,000,000 permutations using PLINK [36] for both allelic (empirical P = 0.079) and genotypic (empirical P = 0.051) models. Assuming perfect linkage disequilibrium between the genotyped and functional SNP and a multiplicative risk model, our sample was 80% powered to detect a heterozygous genotype relative risk of 1.8 with a type I error of 0.5% [37]. The *APOE *ε4 allele is a major risk factor for LOAD therefore we also stratified our samples by presence or absence of the ε4 allele. The minor allele frequency was 5.7% and 5.6% in the ε4 and non-ε4 group respectively. Although not significant, it is interesting to note that both AA individuals were also homozygous for the ε4 allele.

**Table 1:** Statistical analysis of *HECTD2 *marker *rs12249854*

***rs12249854***	**AD n (%)**	**Controls n (%)**	**P-value** **(Chi-square)**	**P-value** **(PLINK)**
AA	2 (0.6)	0 (0.0)	0.0648	0.051
TA	33 (10.3)	47 (7.8)		
TT	285 (89.1)	554 (92.2)		
A	37 (5.8)	47 (3.9)	0.0668	0.079
T	603 (94.2)	1155 (96.1)		

P-values were calculated using a chi-squared test and as an alternative we also implemented 1,000,000 permutations using PLINK [36].

## Discussion

LOAD is a major cause of dementia and is becoming increasingly important in ageing populations. Identifying the genetic susceptibility factors may help to identify those at risk of developing the disease and the effective targeting of future preventative treatments. With the exception of *APOE*, no genes have been conclusively shown to be susceptibility factors for LOAD. In this study, we genotyped a panel of AD patients and looked for an association with a *HECTD2 *SNP previously shown to be associated with prion disease [20]. Although the minor allele frequency was higher in the AD samples than the controls this was not significant. This result suggests that in spite of being a promising candidate, *HECTD2 *is not implicated in AD. Although a negative study, our data do not totally exclude *HECTD2*. Our study assumes that, as for prion disease, a common variant of *HECTD2 *is the susceptibility allele. However, multiple rare variants would not be detectable by our tagging SNP. Re-sequencing of *HECTD2 *in a panel of AD samples would be required to exclude this possibility.

LOAD has a complex aetiology and collections of patient samples may be highly heterogeneous, therefore, very large sample sizes may be required to detect small effects. The heterogeneity of the disease phenotype and the varying inclusion criteria used in different studies may contribute to the difficulties in replicating promising findings from different centres.

Prevalence figures suggest that LOAD affects 20% of people aged 75-84 years and rising in older age groups [1]. No detailed age information was available for our control group, however, samples were taken from current blood donors which suggests that although currently unaffected by dementia it is likely that a significant proportion will develop AD later in life and are therefore likely to carry susceptibility alleles. This may confound our findings. Large cohorts of non-demented elderly individuals are difficult to obtain, however, their use would increase the power to detect genetic susceptibility factors.

Several studies have reported evidence for a LOAD susceptibility locus on Chr 10q, however, the exact locations vary and the regions are frequently broad with added uncertainty about the number of genes involved. It is hoped that results from large, well controlled and replicated GWAS studies will successfully identify the susceptibility alleles on Chr 10q and at other loci across the genome.

## Conclusion

Based on linkage data, association with another neurodegenerative disease and association with the ubiquitin-proteosome system, *HECTD2 *is a promising candidate susceptibility factor for LOAD. In this study we have genotyped a panel of AD patients for the *HECTD2 *SNP *rs12249854 *(A/T) and compared this data to a previously genotyped control population. The minor allele (A) frequency was increased (5.8%) compared to controls (3.9%), however, this was not statistically significant. We were unable to exclude weak or moderate effects on AD risk. No differences were seen when the population was further stratified by *APOE *ε4 genotype. Based on this data we conclude that the common variants of *HECTD2*, as detected by our tagging SNP, are unlikely to be strong susceptibility factors for LOAD in the UK population.

## Competing interests

The authors declare that they have no competing interests.

## Authors' contributions

Research was planned by SEL and JC. Genotyping was carried out by SEL. Patients were assessed and samples provided by MR and NR. Analysis of data was done by SEL and SM. The manuscript was written by SEL, SM and JC. All authors read and approved the final version of the manuscript.

## Pre-publication history

The pre-publication history for this paper can be accessed here:


